# Common Statistical Errors in Scientific Investigations: A Simple Guide to Avoid Unfounded Decisions

**DOI:** 10.7759/cureus.33351

**Published:** 2023-01-04

**Authors:** Alessandro Rovetta

**Affiliations:** 1 Research and Disclosure Division, R&C Research, Bovezzo (BS), ITA

**Keywords:** significance, public health, epidemiology, effect size, common errors

## Abstract

During my experience as an author, peer reviewer, and editor during COVID-19, I have encountered - and committed - various errors related to the interpretation and use of statistical measures and tests. Primarily concerning health sciences such as epidemiology, infodemiology, and public health, the evidence used to inform a conclusion carries an extremely high weight as it translates into decisions made to preserve the population's well-being. Therefore, the aforementioned evidence must be reliable. This short guide discusses the most common and dangerous mistakes I have experienced during my scientific journey. Real and invented examples have been proposed and analyzed in detail, showing possible interpretations, both correct and incorrect, and their consequences. Such a framework makes it clear that a statistical test alone cannot answer any scientific questions. Indeed, the interpretation of results and the verification of assumptions and test eligibility - subject to the author's evaluation - are crucial components of the integrity of the scientific investigation. Before using a test or adopting a measure, we must ask ourselves the following fundamental questions: Are there valid reasons to explore my research question? Am I sure my approach can fully and adequately answer my research question? Am I sure that my model's assumptions - basic and hidden - are sufficiently satisfied? How could violating those assumptions affect the validity of the results and stakeholders? Is the effect size relevant regardless of statistical significance?

## Introduction

During the COVID-19 crisis, between May 2020 and December 2022, I published several peer-reviewed articles, carried out hundreds of peer reviews for over dozens of scientific journals, and edited various manuscripts for a couple of journals. In this experience, I noticed a recurrence of errors (including mine) concerning the use and interpretation of statistical measures and tests. In this regard, numerous authors have extensively addressed similar problems. For example, Greenland et al. thoroughly analyzed various errors in interpreting *P*-values and confidence intervals (CIs) [1]. Nonetheless, in this paper, I try to summarize and explain, most simply, the principal mistakes I pointed out during the last two years. In this scope, I adopt real and realistic health-related examples. In particular, I made this paper to provide a guide to help authors (including myself) develop more robust manuscripts and achieve more solid conclusions. Although correct data and calculations are unlikely to be useless or harmful to the scientific community, misinterpretations of the latter pose a severe threat to public health.

## Technical report

CIs and statistical thresholds

It is too widely believed that overlapping 1 - *α* CIs (e.g., 95% CI) imply *P* > *α* (e.g., *P *> 0.05). Here, we quickly prove that this implication is simply not true. For example, we apply the Welch t-test for the following average values (*N *= 100): *X *= 0 (SD 0.05) and *Y *= 1 (SD 0.5). The 95% CIs are (-0.10, 0.10) and (0.02, 1.98), i.e., they overlap (intersection size 0.08). Nonetheless, the test result is *P *= 0.049 < 0.05 = *α*. It can be proved that nonoverlapping 1 - *α* CIs imply *P *< *α*; however, as extensively discussed in the literature, obsession with significance thresholds should be discouraged (indeed, *P* = 0.049 and *P* = 0.051 are practically identical values) [1].

Statistical significance and effect size

On too many occasions, the existence and relevance of a certain phenomenon are attributed to a related statistical significance. Although this is wrong for various purely theoretical reasons (for instance, statistical significance regards the test adopted and not the phenomenon), here we focus on a more practical aspect: the applicability of a decision made by evaluating only statistical significance. Suppose my cardiologist recommended I lose weight significantly so as to reduce the risk of a heart attack. Thus, I measured my weight weekly for 10 weeks, obtaining the values ​​shown in Table 1.

**Table 1:** Weight in kilograms per week.

Week	1	2	3	4	5	6	7	8	9	10
Weight (kg)	100.5	100.4	100.4	100.3	100.1	100.2	100.1	99.9	100.1	99.8

Applying a linear regression model, I found that the residuals were sufficiently compatible with the hypotheses of distributive normality (Shapiro-Wilk test *P *= 0.67) and constant variance, also known as homoskedasticity (White test *P *= 0.28). As the model was reliable, the slope of -0.07 kg/week (SE 0.01) - which strongly supported the negative trend - was very significant (*P *= 0.0001). Nonetheless, when I presented these highly significant findings to my cardiologist, he was hospitalized for a heart attack due to a fit of anger (sarcasm). Indeed, I believe that, however significant these results were, the 700 g lost in 10 weeks was not the health goal set.

Multicomparison adjustment of *P*-values

Adjustment for multicomparison is often necessary to reduce the number of false positives but introduces the risk of increasing false negatives. Hence, as in the previous case, its implementation must be assessed on a case-by-case basis. For example, if my objective is to detect anomalies that are potentially very dangerous to public health (e.g., via metal detectors in an airport), it is better to have a high number of false positives (false alarms of armed persons) than a high number of false negatives (which is equivalent to not identifying armed individuals). Conversely, if my purpose is to conduct an exploratory investigation (e.g., to look for causal correlations between various theoretically unrelated phenomena), then it can be crucial to avoid the so-called *look elsewhere effect* and reduce (but not eliminate) spurious associations. A real-world example concerning Bonferroni correction is reported in the next section.

Inferential and descriptive statistics

Let's start with a real example, which is the number of annual deaths in Italy from 2015 to 2021 (data taken from ISTAT [1]). Considering that there is high data compatibility with the hypothesis of normality from 2015 to 2019 (Shapiro-Wilk test *P *= 0.54) and we do not detect clear historical trends in that period, we calculate the average number of deaths between 2015 and 2019 (*X*) and compare it with those ​​of 2020 (Y1) and 2021 (Y2). After that, we subtract this average from all dataset values ​​in order to have the number of excess deaths, obtaining the new measures *X *= 0 (SD 13,400, SEM 6,000, range [-19,000; 13,500]), Y1 = 100,000 (95% CI [88,000; 112,000]), Y2 = 63,000 (95% CI [51,000; 75,000]). Grubbs tests for (Y1, *X*) and (Y2, *X*) returned *P *= 0.007 and *P *= 0.04, respectively. Although these numbers are merciless and unambiguous, we now show how choosing an inappropriate test and/or adjustment can produce dangerous false negatives. For instance, implementing the Bonferroni correction (and for only two tested hypotheses), we get *P *= 0.01 and *P *= 0.08. Again, this makes it clear that the evaluation of the effect size must be totally separated from statistical significance and *P*-value adjustment is not always the best choice. Indeed, in the context of public health, the 63,000 excess deaths, taking into account both the narrow CIs and minimal data variability from 2015 to 2019, represent an extraordinarily alarming figure despite its statistical significance. But that's not all - even admitting a larger variability, statistical fluctuations capable of creating such high excesses are a major public health situation (that can lead, for example, to hospitals' overcrowding).

Models' assumptions

**Figure 1:**
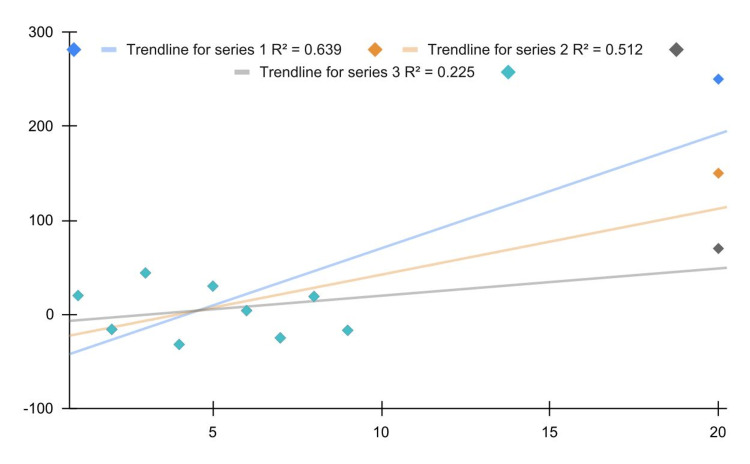
Relationship between Pearson correlation and outliers.

Statistical tests and models are reliable only when their assumptions are sufficiently compatible with our data. Too often, little importance is given to this aspect, which is absolutely essential for drawing valid scientific conclusions. For example, Figure 1 shows three different outliers (blue, orange, and gray dots) for the same dataset (light-blue dots) and the associated linear correlations. As the use of the Pearson correlation standardly requires distributive normality (which implies the absence of outliers), it is evident that these results are biased (if the aim is to find a global linear relationship). The correlation value increases as the outlier increases. The associated *P*-values ​​are 0.005, 0.02, and 0.17.

**Figure 2:**
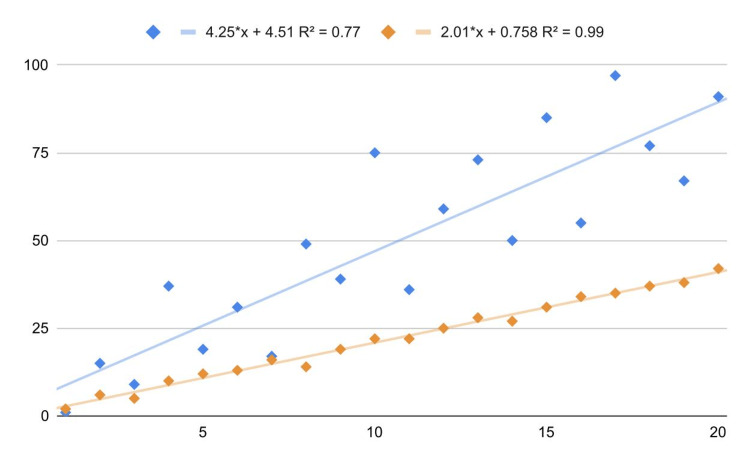
Heteroskedasticity example (blue line) and absence of relationship between correlation and slope.

In this regard, it is important to underline that *P*-values ​​are biased downward as they signal not the correlation significance but the violation of the normality assumption (i.e., they signal that the data are poorly compatible with the test hypothesis assumptions rather than the test hypothesis itself). Another example concerns homoskedasticity in linear regression models: indeed, its violation does not compromise the validity of the model but only the estimation of errors and the *P*-values. This can be seen in Figure 2 (blue line): the data are very compatible with residual normality (Shapiro-Wilk test *P *= 0.48) but not with homoskedasticity (White test *P *= 0.001; the problem could be overcome by applying a weighted regression). Nonetheless, the linear behavior of the data is evident; thus, the model could still be useful for making decisions without considering inference and statistical errors.

Correlation value and slope

Phenomenon and correlation intensity are two different and generally independent aspects. As shown in Figure 2, the correlation value is not related to the function slope: indeed, the blue line has a greater slope (about 4) than the orange line (about 2) despite its R2 being markedly lower (0.77 versus 0.99).

Hypothesis targeting and causation

**Figure 3:**
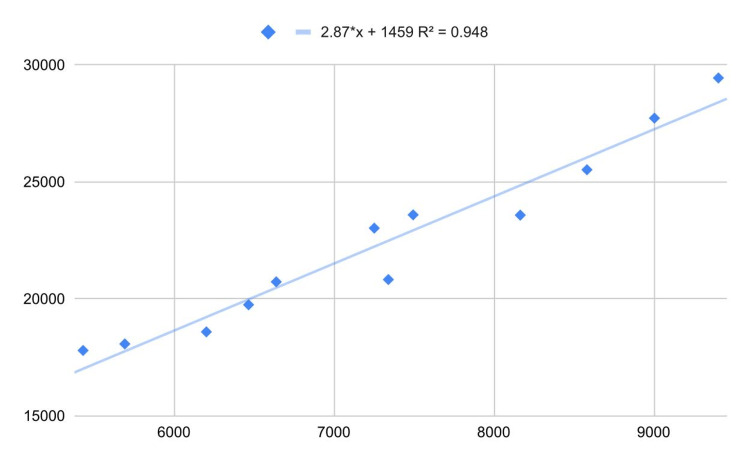
Unknown correlated real data.

Suppose I present the following real data to you without specifying what they represent (Figure 3).

**Figure 4:**
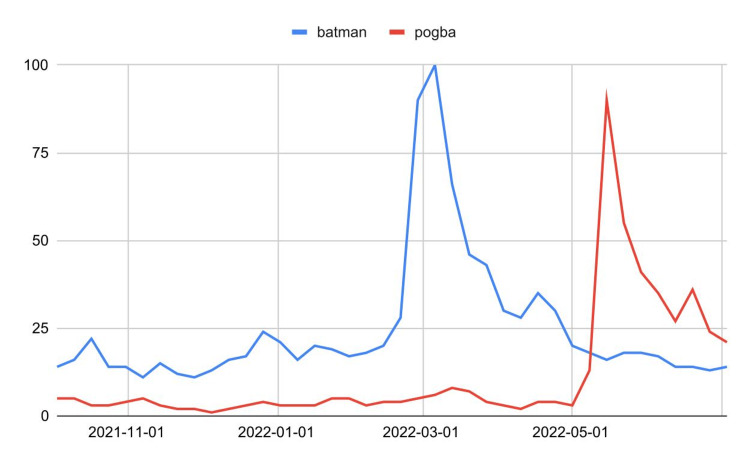
Italian users' web interest in Batman and Pogba.

Applying the usual linear regression model, it turns out that there is little evidence against distributive normality and homoskedasticity (Shapiro-Wilk test *P *= 0.28, White test *P *= 0.21). Furthermore, the correlation is marked (*R *= 0.97, 95% CI [0.91, 0.99]) and highly significant (*P *< 0.0001). It seems reasonable to conclude that at least one of the two variables affects the other or if the correlation is spurious, there is a confounding factor that affects the two variables similarly. Nonetheless, the two variables are the annual US money invested in science, space, and technology and the number of suicides by hanging, strangulation, and suffocation [2]. This shows an obvious but often underestimated fact: statistics alone can never provide evidence of causation. The targeting of a research hypothesis - understood as the set of evidence and reasoning that make a hypothesis worthy of research - is an essential part of any scientific investigation (including exploratory studies). Other misinterpretations concern Granger causality: indeed, Granger causality is not true causality [3]. For example, by searching Google Trends for the queries *batman* (comics superhero) and *pogba* (soccer player) [4], we get the following graph (Figure 4). We apply the Granger causality with time lag = 11 weeks (one degree of nonseasonal differentiation for both series) and find ample evidence against *Y* ≠ *f*(*X*) (*P *= 0.002). Therefore, we should conclude that the web interest in Batman predicts the web interest in the soccer player Pogba, which is very unlikely (Google Trends-related queries confirm the absence of a causal relationship).

CIs and data variability

In many papers, it is customary to report CIs as an estimate of the precision of the measurement/calculation. While they are necessary to assess the relevance of the effect size, confidence (or credible) intervals concern the distribution of the average values; hence, in general, they do not provide information on the dataset variability. Proof of this can be easily obtained from the following example: Suppose we have a Gaussian dataset of 100 elements with an SD of 20 and another Gaussian dataset of 9 elements with an SD of 9. Both datasets have mean values equal to 0. The 95% CIs are (-4, 4) and (-6, 6), respectively. Therefore, the CI of the first dataset is narrower than the second despite its considerably larger SD. Again, we must always ask ourselves what our research purpose is and how our data and analysis characteristics can affect stakeholders.

Central limit theorem

**Figure 5:**
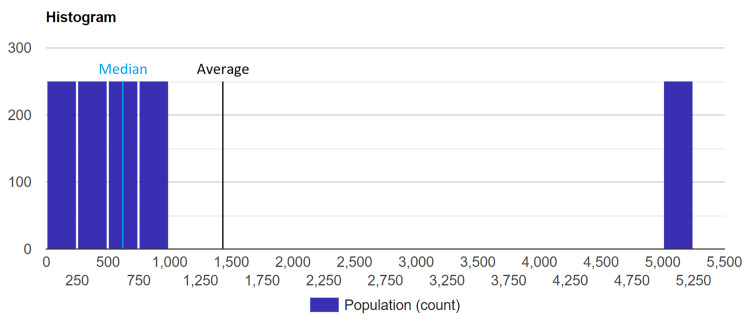
Non-Gaussian large dataset.

The central limit theorem is often invoked to state that a dataset has Gaussian characteristics as it is sufficiently numerous (e.g., *N* > 30). However, this is simply wrong and misleading. Indeed, the central limit theorem concerns only the population of the mean values and not the original population or sample [5]. It follows that it is useful for applying parametric tests, but it does not justify the adoption of descriptive statistics such as mean, variance, or SD for non-Gaussian datasets. In this regard, an easy counterexample can be proposed: considering the dataset of 1250 elements shown in Figure 5, the Shapiro-Wilk test returns *P *< 0.0001. Moreover, the percentage difference between the median and mean is equal to 78.1%. Finally, the average value is outside the dataset. In conclusion, this dataset is extremely non-normal.

Mixed use of different tests: a precautionary note

It is a common practice to use different tests to measure the significance of discrepancies between similar datasets. For example, if the purpose is to compare three pairs of datasets, of which two are normally distributed, and one is not, then some authors may choose two parametric tests and one nonparametric test, respectively. Nonetheless, it is essential to consider that these tests might not only measure different quantities or the same quantity in different ways but that tests' assumptions and the sensitivity to their violation could also be very different. Thus, the same exact *P*-value (e.g., *P *= 0.02) obtained from two different tests could imply a very different degree of statistical significance and meaning (of course, this could also happen even when the same test is applied multiple times, but the chance is lower according to what discussed above). Consequently, when the purpose is to inform a single set of decisions, adopting mixed tests could create a heterogeneity capable of strongly biasing the results. In this regard, considering these aspects in the analysis and the related uncertainty in the final conclusion is imperative.

Counterfactual scenarios

**Figure 6:**
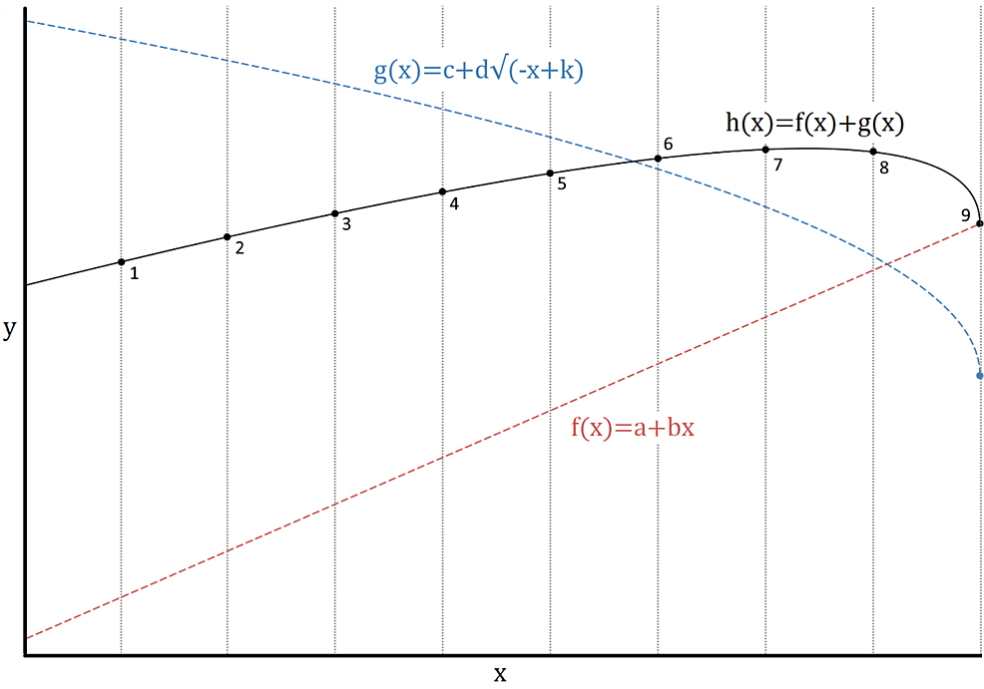
Example of a statistical anomaly due to the superposition of an increasing linear trend and a decreasing sublinear trend. Indeed, measure number nine is very far from the prediction of a model built or trained on the first eight values. However, such an anomaly is not due to any external factor (i.e., the reason is endogenous). Source: [6]

Evaluating a counterfactual scenario is one of the most complicated and important tasks in science. For example, if the objective is to estimate the impact of COVID-19 on mortality, it is necessary to make a prediction - without COVID-19 - based on the previous historical trend (e.g., the prior 5 or 10 years). This assessment is entirely based on the assumption that a trend remains unchanged in the absence of significant external events (the principle of parsimony). While this assumption is reasonable, and the history of science confirms its validity, a moderate degree of uncertainty must be considered. For instance, the superposition of nonlinear trends could give rise to a linear trend up to a certain date (threshold) and a nonlinear trend beyond the latter (Figure 6). If the external event occurs in conjunction with the threshold, an improper causal association could be asserted (this is not the case with COVID-19 impact on mortality [6]).

Moreover, the external factors could be multiple, which makes it difficult to identify which of these was predominant for the change in trend. For these reasons, once again, the scientific targeting of the hypotheses is fundamental for the investigation's validity. Indeed, only the combination of evidence from various disciplines can provide solid conclusive evidence. On the other side, this exposes the research more to authors' bias [7]. Finally, it is possible to obtain different results by observing different temporal windows to make comparisons. For example, the statistics on excess deaths in Italy during 2020 are slightly different if we consider the previous five years as a comparison time-lapse instead of the previous 10 years [6]. This happens because all the real models have a margin of uncertainty, and it is not always easy to select the correct width of the temporal window.

Hidden assumptions

**Figure 7:**
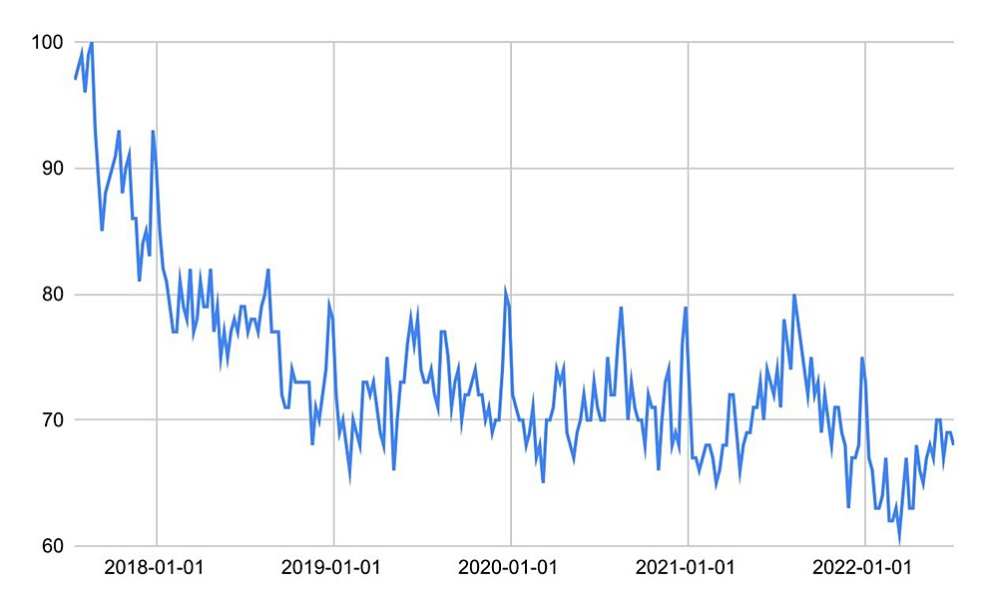
Italian users' web interest in porn.

Suppose we need to measure the impact of the COVID-19 pandemic on web interest in porn [8]. Thus, exploiting the central limit theorem, we apply a Welch t-test between the 2017-2019 and 2020-2022 datasets, obtaining a high (7.4, 95% CI 6.0-8.9) and significant (P<.0001) negative decrease due to COVID-19. Since the test's assumptions have been met, it could be concluded that the result is reasonable. However, comparing statistics such as mean and median requires a fundamental implicit assumption that there are no trends and seasonality in the time series (i.e., there is sufficient stationarity). Indeed, an evident downward trend was already underway before COVID-19 (Figure 7). Nevertheless, while the result found is not surprising despite its low *P*-value (i.e., it is in line with the predictions or, at best, cannot answer the research question), it is still helpful to support the presence of a historical decreasing trend and quantify the global decrease.

Wrong choice of test for the goals of analysis

Suppose we want to measure the change in average daily particulate matter (PM) 2.5 concentrations during two specific weeks of two consecutive winters in a certain area. For this example, we assume that no historical trends were in place. Data were considered sufficiently normal (Shapiro-Wilk test *P* = 0.35) to adopt the one-sample t-test on the paired differences (Table 2).

**Table 2:** An example of average daily PM 2.5 concentrations (μg/m3) during two specific weeks of two consecutive winters in a certain area. *Critical value for public health. CI, confidence interval; PM, particulate matter

Day	1	2	3	4	5	6	7	8	9	10	11	12	13	14	*P*-value	95% CI
2020	38^*^	41^*^	16	29^*^	13	26^*^	15	21	14	13	27^*^	18	10	25^*^	0.09	-1; +inf
2019	23	16	19	20	19	17	23	15	20	14	21	15	18	14		

The test result provides little evidence against the difference in the paired differences of PM 2.5 concentrations (*P *= 0.09). Hence, the two distributions could be considered similar. Nonetheless, we must ask ourselves: Are we applying the proper test for this public health assessment? The answer is no as exceeding some critical PM 2.5 concentrations ​​poses a more serious threat to health [9]. Indeed, the safety values ​​were exceeded in 6 out of 14 days in the 2020 series. In this regard, the negative lower limit of the 95% CI suggested that the test had poor accuracy due to the high variance of 2020 (CV1 = 43% versus CV2 = 17%). Another known but noteworthy case is that of the Mann-Whitney U test, which is frequently used to measure the significance of the difference between two medians or distributions. As has also been shown in other literature, this can be wrong in many ways [10]. Indeed, the Mann-Whitney test ranks all the values from low to high and then compares the mean ranks; therefore, it is sensitive to data spread [11]. It can be used to compare medians (and even means) only when the distributions of the two populations have the same shape. Thus, in this case, it is not a matter of the test’s assumptions but the analysis' goal.

Comparison of results

Suppose we have two independent samples of *N *= 10 elements that sufficiently meet all the assumptions for using the Pearson correlation properly. The two correlations have values *x *= 0.70 (95% CI 0.13-0.92; *P* = 0.02) and *y *= 0.50 (95% CI -0.19 to 0.86; *P *= 0.14). Based on these *P*-values, we conclude that the first correlation is much more significant than the second. This could lead us to say there is a substantial difference between the two correlations. Nevertheless, comparing their values ​​with the method of Eid et al. [12], we get *P *= 0.28. Therefore, as also underlined by the large CIs, there is little evidence against the equality of the two correlations. This shows that to make claims about the comparison of two (or more) results, we must directly compare them through an appropriate test (or evaluate their effect size) and not rely on their single statistical significance.

Multicomparison adjustment to inform a final decision

One of the most common errors is to believe that the adjustment for the multicomparison should only be applied when the results or data are directly compared with each other (e.g., as done in the Comparison of Results section). Indeed, the adjustment for the multicomparison - when deemed appropriate - should be made when the final decision (e.g., to apply a lockdown based on the abnormal increase in flu symptoms among a population) is based on the result of multiple tests, regardless of whether these tests are mutually comparative or independent [6]. As a matter of fact, the increased likelihood of false positives is due to the high number of tests used and not to the nature of the latter [13].

Presentation of results

As also done in this paper, we often talk about test results as if they were properties of the dataset (e.g., the values ​​are normally distributed). This approach is technically inaccurate as applying different tests could produce different results. Indeed, the *P*-value is related to the test adopted and not the dataset itself. To prove this, we consider the following vector *X *:= (-14, 0, 1, 2, 3, 5, 6, 7, 9). If we apply the Shapiro-Wilk test to its components, we obtain *P *= 0.03, which suggests that the dataset is not normal (i.e., there is low compatibility with the normality hypothesis). On the contrary, if we apply the Kolmogorov-Smirnov test, we obtain *P *= 0.51, which suggests that the dataset can be considered normal (i.e., there is high compatibility with the normality hypothesis). However, this slight terminology abuse could be tolerated as long as the test adopted to evaluate the dataset compatibility with the fixed hypothesis is clearly specified.

## Discussion

This brief overview provides practical indications for epidemiological analysis and public health surveillance strategies. It has been proven that overlapping CIs do not imply exceeding the significance threshold (while it is true that nonoverlapping CIs imply *P*-values ​​lower than the threshold). However, as *P*-values are graded measures, the adoption of significance thresholds should be discouraged. Statistical significance, when properly used, provides information on how compatible (*P*-values) or surprising (*S*-values = -log(*P*-value, 2)) the results found are compared to the test hypothesis adopted through the chosen test, but, in general, it does not provide information on the effect size. Therefore, the intensity of a phenomenon or the importance of an outcome must be assessed separately according to the scientific goal, context, and stakeholders (especially when public health is involved). Adjustment for multicomparison should be evaluated according to the scientific or even ethical relevance of the decision to be informed (e.g., conservation of public health). Comparing two or more results based on their single significance is unreliable and potentially very misleading, as only a comparative test or a specific evaluation of their effect sizes provides information on their mutual relationship. If considered appropriate, the adjustment for multicomparison should only be applied based on the number of tests used to inform the final scientific decision and not on the type of test performed. In particular, it is not necessary for the test to directly compare two or more outcomes or data to require adjustment for multiple comparisons. Descriptive and inferential statistics offer different clues to inform public health or any other scientific decision. In particular, inferential statistics do not always fit into the context of public health surveillance. The metal detector approach should be enforced during health crises (especially when the available data is little) to counter or prevent their effects. Verifying the models' assumptions and evaluating the effects due to their violation is crucial to draw scientifically valid conclusions. Reviewers and editors of all academic journals and agencies should specifically request a thoughtful description of how these aspects were managed in a manuscript. Assuming that all the appropriate assumptions are verified, the correlation value provides information on how well the data is positioned along a line (e.g., Pearson) or other function (e.g., Spearman). However, the slope of these functions is a different and generally independent aspect. The hypothesis targeting is the basis of any scientific exploration; indeed, no statistical analysis (including Granger causality or similar) can provide evidence of causation without the latter. CIs and credibility intervals provide information on the distribution of mean values ​​but not on the variability of the dataset. This aspect must be considered according to the purpose of the analysis. Furthermore, it is always necessary to ask which characteristics of our data are relevant to inform the final decision and stakeholders. The central limit theorem guarantees the normality of the distribution of the mean values ​​of a sufficiently large dataset but is in no way valid for the dataset itself. The dataset's characteristics must be evaluated using appropriate tests and graphical representations according to the research purpose. The test used in this scope must always be clearly specified since *P*-values regard the test and not the dataset. Mixing different types of statistical tests to analyze similar datasets can lead to marked heterogeneity of scenarios, which can compromise the validity of the conclusion based on these tests. Carefully evaluating this aspect is fundamental to guarantee the scientific integrity of the whole research and inform the final decision properly. The evaluation of a counterfactual scenario is complex and always subject to a nonnegligible margin of unavoidable uncertainty. Therefore, hypothesis targeting and scientific rationale are key factors in determining a counterfactual model's validity and plausibility. When we use a statistical test, we don't just have to verify its standard assumptions but also ask ourselves if our data are suitable for applying such a test to respond to our research question. Moreover, when we use a statistical test, we must adequately consider whether this is suitable to help us in responding to our research question. Indeed, even if all assumptions (both primary and hidden) are verified, mere statistics cannot provide solid evidence for or against its use for a given scientific objective.

## Conclusions

This framework makes it clear that a statistical test alone cannot answer any scientific questions. Indeed, the interpretation of results and verification of assumptions and test eligibility - subject to the author’s evaluation - are crucial components of the integrity of the scientific investigation. Therefore, before using a test or adopting a measure, we must ask ourselves the following fundamental questions: Are there valid scientific reasons to explore my research question? Am I sure my approach can fully and adequately answer my research question? Am I sure that my model's assumptions - basic and hidden - are sufficiently satisfied? How could violating those assumptions affect the validity of the results and the stakeholders? Is the effect size relevant regardless of statistical significance?
